# Investigating the Molecular Processes behind the Cell-Specific Toxicity Response to Titanium Dioxide Nanobelts

**DOI:** 10.3390/ijms22179432

**Published:** 2021-08-30

**Authors:** Laurent A. Winckers, Chris T. Evelo, Egon L. Willighagen, Martina Kutmon

**Affiliations:** 1 Department of Bioinformatics—BiGCaT, NUTRIM School of Nutrition and Translational Research in Metabolism, Maastricht University, NL-6200 MD Maastricht, The Netherlands; laurent.winckers@maastrichtuniversity.nl (L.A.W.); chris.evelo@maastrichtuniversity.nl (C.T.E.); egon.willighagen@maastrichtuniversity.nl (E.L.W.); 2 Maastricht Centre for Systems Biology (MaCSBio), Maastricht University, NL-6200 MD Maastricht, The Netherlands

**Keywords:** nanomaterials, titanium dioxide, nanobelts, overrepresentation analysis, Gene Ontology, THP1, SAE, Caco-2/HT29-MTX

## Abstract

Some engineered nanomaterials incite toxicological effects, but the underlying molecular processes are understudied. The varied physicochemical properties cause different initial molecular interactions, complicating toxicological predictions. Gene expression data allow us to study the responses of genes and biological processes. Overrepresentation analysis identifies enriched biological processes using the experimental data but prompts broad results instead of detailed toxicological processes. We demonstrate a targeted filtering approach to compare public gene expression data for low and high exposure on three cell lines to titanium dioxide nanobelts. Our workflow finds cell and concentration-specific changes in affected pathways linked to four Gene Ontology terms (apoptosis, inflammation, DNA damage, and oxidative stress) to select pathways with a clear toxicity focus. We saw more differentially expressed genes at higher exposure, but our analysis identifies clear differences between the cell lines in affected processes. Colorectal adenocarcinoma cells showed resilience to both concentrations. Small airway epithelial cells displayed a cytotoxic response to the high concentration, but not as strongly as monocytic-like cells. The pathway-gene networks highlighted the gene overlap between altered toxicity-related pathways. The automated workflow is flexible and can focus on other biological processes by selecting other GO terms.

## 1. Introduction

Engineered nanomaterials have become important in our daily life as they are utilized in the fields of food, packaging, cosmetics, drug and vaccine delivery, and many others [1]. For example, silver and carbon nanotubes are used in a variety of cleansers because of their antimicrobial properties, and silicon dioxide is used as a food additive as it decreases viscosity and regulates acidity [2]. Nevertheless, some nanomaterials, such as asbestos fibers and silica dust, show how small particles can cause adverse outcomes for those exposed to them [3,4,5,6].

The detailed biological processes related to the toxicity of many engineered nanomaterials are not yet all fully understood [7,8]. Due to the varied physicochemical properties of nanomaterials and often even of the particles within the nanomaterial itself, it is difficult to predict the biological effects leading to toxicity. Biological response and toxicity depend on the size of the nanoparticle, size of the agglomerate, surface impurities, and degradability [9]. Furthermore, other factors that have an effect on biological response and toxicity include the method of exposure, the entry route into the human body, and the distribution in the body [2]. The shape of a nanoparticle also has an influence on the nanoparticle’s effects, where for example nanobelts, nanostructures in the form of belts, have been shown to be more pro-inflammatory compared to spheres [10]. Studies have found that there are various biological effects and toxicities of engineered nanomaterials under different circumstances and with varying engineered nanomaterials [1,11,12,13,14]. These changes in biological effects are ascribed to the differences in chemical and physical properties of nanomaterials. However, when studying the properties, biological effect relations are complicated because of the difficulty in creating identical nanomaterials from different batches and/or sources due to the varying physicochemical properties of nanomaterials [15]. Nevertheless, manual curation of transcriptomics datasets regarding nanomaterials is performed to enhance their degree of fairness to aid nanomaterial safety assessment [16].

Furthermore, a relatively well-studied and widely used nanomaterial is titanium dioxide (TiO_2_). Due to its general properties, such as its photocatalytic activity and white color, it is used in various applications such as (photo)catalysis, antibacterial agents, and consumer products. Whereas TiO_2_ particles have been shown not to be able to penetrate through the skin, entry into the body can occur via inhalation or ingestion where it then has to pass through the gastrointestinal tract [17,18].

Regarding the adverse outcomes, the toxic effects of TiO_2_ are known to occur in order, like most toxicity related biological processes. TiO_2_ is known to induce reactive oxygen species production, which involves lipid peroxidation and will eventually lead to cell damage and DNA damage [19,20]. Furthermore, the increase in oxidative stress can contribute to the promotion of apoptosis of the affected cells [21,22]. Consequently, TiO_2_ nanoparticles have been shown to induce inflammation [23,24] among other things via the aforementioned oxidative stress in mammalian cells [19,25]. It has been shown that TiO_2_ nanoparticle exposure can lead to impaired immune homeostasis including increases in TNF-α, IFN-γ, IL-2, IL-4, IL-6, IL-8, and IL-10 secretion [26,27,28].

To study the detailed mechanisms of the cellular response, bioinformatics and systems biology approaches, including pathway and network analysis, have been used to assess the effects on toxicity-related processes upon exposure to TiO_2_-nanobelts, i.e., reactive oxygen species formation and oxidative stress, apoptotic cell death, inflammation, and DNA damage [29,30]. Pathway enrichment analysis helps to put high-throughput omics data such as transcriptomics into a biological context [31] and the visual diagrams from pathway databases such as WikiPathways [32] and Reactome [33] provide a way to visualize the effects on cellular processes. However, navigating all the affected pathways and the roles of the genes in these pathways can be nontrivial: genes can participate in multiple pathways, and pathways tend to overlap with each other. Moreover, we want to be able to focus on detailed, molecular pathways related to a specific biological process, such as apoptosis, inflammation, DNA damage, and oxidative stress. Overrepresentation, with respect to these processes alone, makes it possible to focus on a subset of genes, but it does not have the link to the pathways. Instead, we selected pathways based on their gene overlap with specific GO-terms covering toxicity-related biological processes and subsequently used the overrepresentation of differentially expressed genes to select relevant pathways out of this subset. We further studied these results in detail using network analysis approaches to investigate pathway overlap and crosstalk.

In this study we re-examined a publicly available dataset generated by Tilton et al. [34] (accession number: GSE42069) [35] to study the detailed molecular mechanisms of TiO_2_-nanobelt toxicity. We analyzed gene expression data from three different cellular models exposed to either one of two concentrations of TiO_2_-nanobelts for 24 h. The cell lines used were human primary small airway epithelial (SAE) cells, human monocytic cells (THP1), and human epithelial colorectal adenocarcinoma cells co-cultured with HT29-MTX goblet cells (Caco-2/HT29-MTX). These cellular models represent three common areas of exposure in the human body, i.e., small airway epithelium, colon epithelium, and the innate immune system that will typically respond to particles that have entered the body. We aim to provide an overview of the dose-dependent and cell-type-specific response by focusing on toxicity-related processes. Apart from a significant increase in the activity of toxicity-related processes after exposure with a higher dosage, differences in intensity and affected processes might occur between the cell lines. Using this work as an example, we will discuss the toxicological effects of TiO_2_-nanobelts on the three cellular models and the importance and benefits of analysis workflows in the nanomaterial research field.

## 2. Results and Discussion

### 2.1. Differential Gene Expression

**Figure 1:**
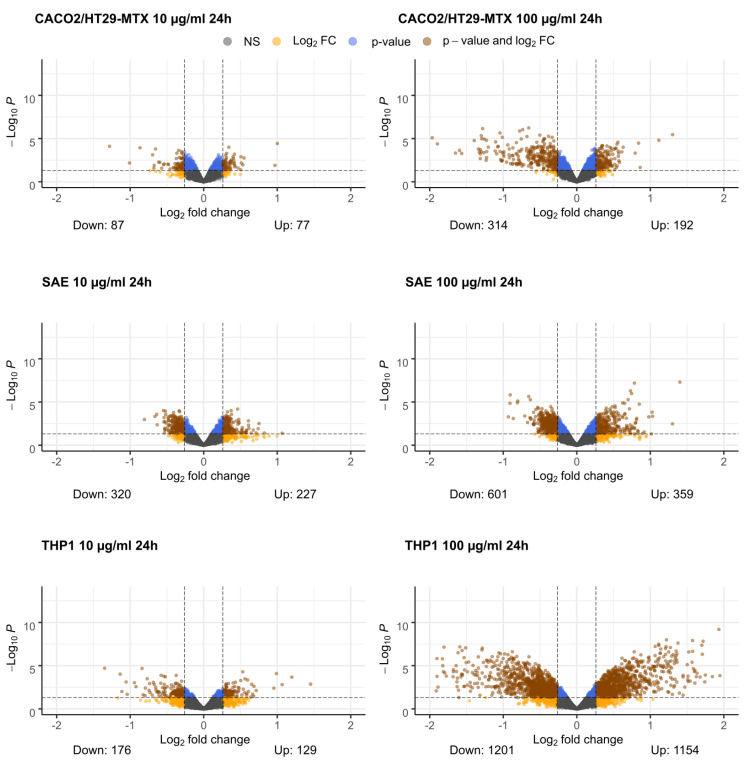
Gene expression volcano plots for different cellular models and TiO_2_-nanobelt concentrations. On the x-axis log_2_ (fold change) is depicted whereas on the y-axis the -log_10_ (*p*-value) is depicted. The dotted lines represent cut-off values for significantly changed genes (log_2_ fold change > 0.26 or <−0.26, *p*-value < 0.05). A brown color depicts that a gene meets both cut-off criteria, a blue color relates to meeting only the *p*-value cut-off, an orange color relates to meeting only the log${}_{2}$ fold change cut-off, and a gray color indicates that a gene does not meet any of the criteria.

The differential gene expression analysis for the three cell lines after exposure to two different TiO_2_-nanobelt concentrations shows that a high concentration of TiO_2_-nanobelts (Figure 1, right column) causes stronger gene expression changes in all three cellular models than at a low concentration (Figure 1, left column). While the Caco-2/HT29-MTX and SAE cells also show an increased response to the high TiO_2_-nanobelt concentration, the THP1 cells respond much more strongly to the high concentration in terms of differentially expressed genes passing our criteria. Dose-dependent increases in response have been shown for many nanomaterials [36,37].

### 2.2. Pathway Analysis

Using the differentially expressed genes from the different cellular models, an overrepresentation analysis was performed to identify altered pathways after TiO_2_-nanobelt exposure. The number of significantly overrepresented pathways (*p*-value < 0.05) is shown in Table 1 in the column “Significant”. Concordant to the increase in differentially expressed genes matching our criteria shown in Figure 1, the number of resulting pathways increases with an increase in the concentration of TiO_2_-nanobelts. While there was a smaller increase in differentially expressed genes, we found a similar increase in resulting pathways for the SAE cell line. There was also an increase in the number of genes found for the Caco-2/HT29-MTX co-culture; however, it did not directly translate to an increase in overrepresented pathways. Although overrepresentation analysis provides a great overview of all processes that are affected, it takes time to go manually over the many overrepresented pathways. Therefore, an automated method to select desired pathways, i.e., toxicity-related pathways, provides a new approach to interpreting the data.

### 2.3. Toxicity-Related Pathways

**Figure A1:**
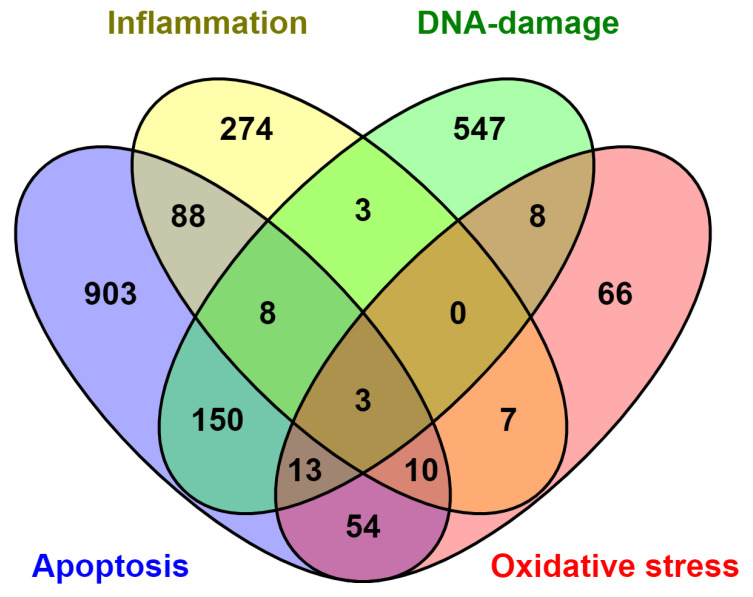
Venn diagram showing the number of overlapping genes between the four GO-terms “apoptotic process”, “inflammation”, “cellular response to DNA damage stimulus”, “DNA damage and Oxidative stress”.

To gain more insight into the toxicity-related processes, the overrepresented pathways were further categorized into pathways related to the apoptotic process, inflammatory response, DNA damage response, and/or oxidative stress. Often this kind of clustering of the pathways is performed manually. However, we implemented a gene-based approach. First, gene sets of four Gene Ontology (GO) terms were obtained, i.e., “apoptotic process” (GO:0006915, 1269 genes), “inflammatory response” (GO:0006954, 569 genes), “cellular response to DNA damage stimulus” (GO:0006974, 762 genes), and “response to oxidative stress” (GO:0006979, 243 genes). Evidently, these processes are tightly connected. Whereas some can be causative for others, they are expected to overlap. Additional Figure A1 shows the gene overlap between the four GO gene sets in a Venn diagram. Next, we calculated the gene overlap between the pathways from the WikiPathways pathway collection with the annotated genes of the four GO-terms. The overlap was calculated by dividing the number of toxicity-related genes present in the pathway by the total number of genes present in the pathway. As a cut-off we used 50% indicating that at least half of the pathway is directly linked to the GO-term of interest via their gene overlap.

To select the desired gene overlap cut-off, we compared four cut-offs with each other and an overrepresentation analysis approach to see how many pathways these would give. The cut-offs we used were 50%, 60%, 70%, and 80%. For the overrepresentation analysis we looked for overrepresentation of the pathway genes in the gene lists of the GO-terms. The results are shown in additional Table A1. The overrepresentation analysis (*p*-value < 0.05) showed the most pathways to be linked to the four GO-terms; in this case it showed 401, 250, 203, and 205 pathways overrepresented for the apoptotic process, inflammatory response, DNA damage, and oxidative stress GO-terms, respectively. The 80% and 70% gene overlap resulted in almost similar amounts of pathways compared to each other. They even resulted in 0 pathways for the inflammatory response GO-term. Moreover, it stands out that for the gene overlap approach only one pathway meets at least 50% or higher gene overlap with the oxidative stress GO-term. Additionally, it is interesting to see that an overrepresentation analysis prompts hundreds of pathways compared to just dozens when looking at gene overlap. Comparing the results of the overrepresentation analysis and various cut-offs, the at least 50% gene overlap cut-off was deemed best for our approach.

After this filtering step we found that, out of the 1076 pathways in the human pathway collection, 66 pathways are related to the apoptotic process, 15 are linked to inflammatory response, 28 are linked to DNA damage, and 2 are linked to oxidative stress. It is noticeable that there are only two oxidative stress pathways, i.e., Detoxification of Reactive Oxygen Species (wikipathways:WP2824 [38]) and Oxidative Stress (wikipathways:WP408 [39]), that have at least 50% of their genes overlapping with the genes annotated to the GO-term “response to oxidative stress”. Based on this finding we can argue that most pathways in the pathway database we have used describe different processes that result in oxidative stress or where oxidative stress is part of/has an influence on the process itself. However, these do not describe oxidative stress as a process. Nevertheless, these pathways are of interest for our analysis.

### 2.4. Study Effect on Toxicity-Related Pathways

The number of altered toxicity-related pathways is shown in Table 1. The overrepresentation analysis was performed using the differentially expressed genes (log${}_{2}$ fold change lower than −0.26 or greater than 0.26 and *p*-value lower than 0.05).

The Caco-2/HT29-MTX co-culture shows for both concentrations 10 overrepresented pathways. The SAE cells show 3 for the low concentration, which could be an indication of a very small toxic response to the TiO_2_-nanobelt exposure, and 15 for the high concentration. The THP1 cell line shows a clear toxic response activation for the high concentration with 39 overrepresented pathways, and 10 for the low concentration.

**Figure 2:**
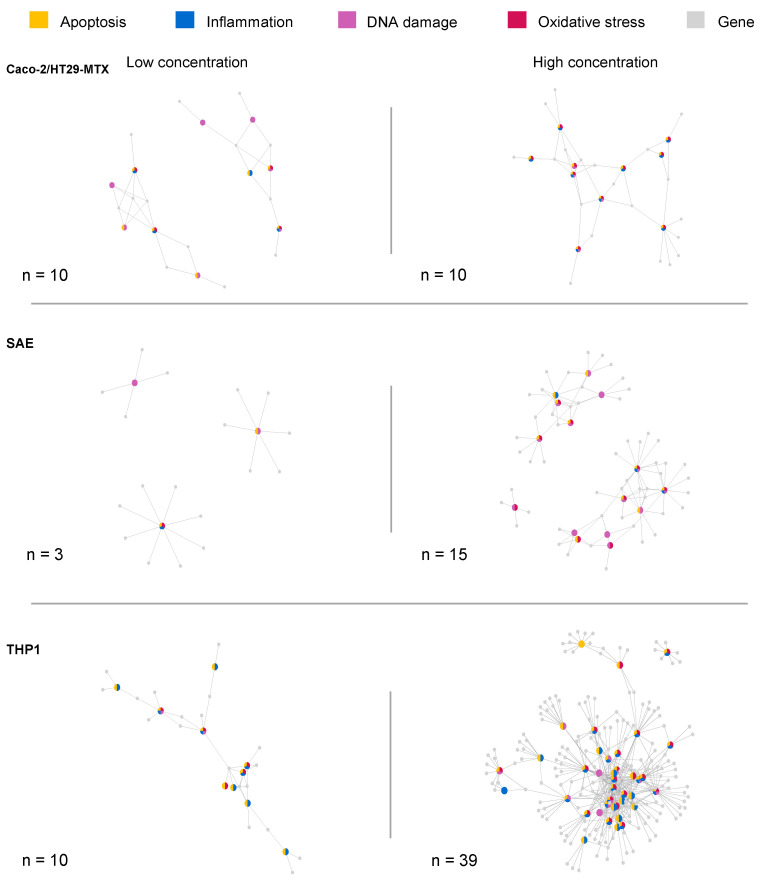
Pathway-gene networks of altered toxicity-related pathways. The color of the nodes indicates to which GO-term the pathway is affiliated. Orange indicates apoptosis, blue indicates inflammation, pink indicates DNA-damage, and red indicates oxidative stress. Gray nodes represent genes. Number (n) represents the number of significantly overrepresented pathways that are depicted in the networks.

Importantly, while molecular pathways describe a process on a detailed level, their boundaries are often not clearly defined. Pathways are not independent, and they interact with each other through shared genes or sub-pathways. Figure 2 shows the gene overlap between the altered toxicity-related pathways in pathway-gene networks and highlights the differences in response between the cell lines. Pathways that cluster together indicate that these pathways depict similar biological processes with a high gene overlap. In the following sections, the biological pathways affected in the different cell lines will be discussed in detail.

### 2.5. Caco-2/HT29-MTX Cells

While for both concentrations the Caco-2/HT29-MTX co-culture shows the same number of overrepresented pathways, the pathways compared between the low and high concentrations are not all the same. Of the ten pathways, three are found for both concentrations, namely IL-5 Signaling pathway (wikipathways:WP127, [40]), IL-2 Signaling pathway (wikipathways:WP49, [41]) and Endometrial Cancer (wikipathways:WP4155, [42]). Interestingly, for the low concentration the pathways related to DNA-damage and repair show up in the results. For example, HDR Through Homologous Recombination (HRR) or Single Strand Annealing (SSA) (wikipathways:WP3567, [43]), Nonhomologous End-Joining (NHEJ) (wikipathways:WP3550, [44]), and DNA Double-Strand Break Response (wikipathways:WP3543, [45]) are among these pathways. However, for the high concentration it is interesting to see that the Apoptosis pathway (wikipathways:WP254, [46]) shows up, which could be an indication of Caco-2/HT29-MTX cells possibly undergoing apoptotic processes upon exposure to TiO_2_-nanobelts. However, these results indicate that the studied processes in Caco-2/HT29-MTX co-cultures are not extensively affected by the administration of TiO_2_-nanobelts, for either the low or the high concentration. This finding supports the conclusion based on the cell viability assay results in the original study, which showed that there was no significant decrease in cell viability for the Caco-2/HT29-MTX co-culture for both concentrations of TiO_2_-nanobelts [34].

### 2.6. SAE Cells

For the SAE cell line, the low concentration only prompted three overrepresented pathways compared to 15 for the high concentration. Noticeable as well, only one of those three pathways was found to be overrepresented also for the high concentration, namely HDR through Homologous Recombination (HRR) or Single Strand Annealing (SSA) (wikipathways:WP3567, [43]). This increase in pathways with a significantly increased overrepresentation of affected genes is an indication that upon exposure to the high concentration of TiO_2_-nanobelts, more biological processes related to toxicity are affected compared to the low concentration.

Next, for the SAE cell line exposed with the low concentration, we found three overrepresented pathways. As for the high concentration we found 15 overrepresented pathways. This increase in pathways with a significantly increased overrepresentation of affected genes is an indication that more biological processes related to toxicity are affected compared to the low concentration upon exposure to the high concentration of TiO_2_-nanobelts. For the low concentration the pathways HDR through Homologous Recombination (HRR) or Single Strand Annealing (SSA) (wikipathways:WP3567, [43]), IL-6 Signaling pathway (wikipathways:WP364, [47]), and Regulation of TP53 Activity through Phosphorylation (wikipathways:WP3838, [48]) are overrepresented. The first pathway describes a process of DNA double-strand break repair, where the second pathway describes the signaling of the cytokine IL-6 and the last pathway describes the regulation of TP53. Except for the first pathway, which is also found in the result for the high concentration, the overrepresented pathways describe more general regulatory pathways rather than detailed process-describing pathways. However, for the high concentration we see multiple pathways related to DNA damage, such as the aforementioned HDR through Homologous Recombination (HRR) or Single Strand Annealing (SSA) (wikipathways:WP3567, [43]), DNA IR-damage and Cellular Response via ATR (wikipathways:WP4016, [49]), DNA IR-Double Strand Breaks (DSBs) and Cellular Response via ATM (wikipathways:WP3959, [50]), and DNA Mismatch Repair (wikipathways:WP531, [51]) among others. Moreover, the results prompted apoptosis-related pathways such as Apoptosis Modulation and Signaling (wikipathways:WP1772, [52]) and Apoptosis Modulation by HSP70 (wikipathways:WP384, [53]). This is also an indication that the high concentration induced more distinct biological processes related to toxicity compared to the low concentration for the SAE cell line.

### 2.7. THP1 Cells

For the THP1 cell line, there are only 10 significantly overrepresented pathways for the low concentration of TiO_2_-nanobelts. However, among these pathways, the pathways TNF Signaling (wikipathways:WP3380, [54]), Apoptosis (wikipathways:WP254, [46]), Apoptotic Execution Phase (wikipathways:WP1784, [55]), TNF alpha Signaling Pathway (wikipathways:WP231, [56]), and TNF-related Weak Inducer of Apoptosis (TWEAK) Signaling pathway (wikipathways:WP2036, [57]) are present. This indicates that upon exposure to the low concentration of TiO_2_-nanobelts the THP1 cell line activates immune- and inflammation-related processes. This result can be explained as a general cell activity response since THP1 is a macrophage-like cell line. However, it can also be explained due to the effect of TiO_2_-nanobelts on the THP1 cell line in this case. TiO_2_-nanobelts are likely to induce toxic processes, even at a low concentration, which results in an inflammatory response of the THP1 cells. Similar inflammatory responses, like activation of Nf-κB and production of TNF-α, were seen in these cells upon exposure to other nanoparticles such as ZnO NM-110, SiO_2_ NM-200, and Ag NM-300 [58].

Compared to the low concentration, the high concentration yields more pathways with significant overrepresentation i.e., 39 versus 10. Except for the pathway Apoptotic Execution Phase (wikipathways:WP1784, [55]) all other 9 pathways which showed up for the low concentration were found for the high concentration as well. In addition to these pathways, pathways such as Oxidative Stress (wikipathways:WP408, [39]), Nanoparticle Triggered Regulated Necrosis (wikipathways:WP2513, [59]), and Mismatch Repair (wikipathways:WP3381, [60]) and more were among the significant pathways. These results indicate that the THP1 cell line upon exposure to the high concentration of TiO_2_-nanobelts results in toxicity-related processes such as inflammation, DNA damage, and oxidative stress. The higher number of pathways that show up in the result together with the biological processes they depict indicate that the high concentration induces greater effects than the low concentration. This was also seen in the original paper where the low concentration induced a significant decrease in cell viability, while the high concentration induced an even greater decrease [34]. Furthermore, it has also been shown that nanoparticles can activate similar processes, such as inflammatory processes and DNA damage, as seen upon exposure to the high concentration [58,61].

The THP1 cell line is the cellular model that has the most inflammation-related pathways in the results: four for the low concentration and nine for the high concentration. All four of the low concentrations show up in the results of the high concentration as well. These pathways are Photodynamic-therapy-induced NF-kB Survival Signaling (wikipathways:WP3617, [62]), Interleukin-10 Signaling (wikipathways:WP4063, [63]), Fibrin Complement Receptor 3 Signaling Pathway (wikipathways:WP4136, [64]), and Platelet-mediated Interactions With Vascular and Circulating Cells (wikipathways:WP4462, [65]). Nanoparticle-induced genotoxicity can arise and a distinction between primary and secondary genotoxicity can be made. Inflammation drives secondary toxicity as suggested by Emma Åkerlund et al., who showed that conditioned media from differentiated THP1 cells induced DNA-damage in HBEC after 3 h exposure [66]. While there are inflammatory-related pathways in the results for the THP1 cell line, only the high concentration has three DNA-damage-related pathways in the results. The presence of DNA-damage-related pathways and inflammatory-related pathways could indicate secondary genotoxicity for the THP1 cells upon exposure to the high concentration. However, more research is needed to address this exact mechanism.

### 2.8. Comparison between Cell Lines

The focused analysis of alterations in toxicity-related processes showed differences between the three cell lines and the concentrations studied. It has been reported before that the molecular response depends on the cell type and concentration [67].

Caco-2/HT29-MTX cells seem resilient to the exposure and show very little activation of toxicity-related processes. While for this dataset a co-culture was used, the passage of nanoparticles through the cellular barriers of Caco-2 cells has been shown to be limited, which could result in reduced uptake and therefore less toxic response [68]. This could partially explain the resilience we see in our results. Subsequently, this indicates a lower importance of gastro-intestinal uptake in general but no direct conclusions could be made based on the results and information we have. However, TiO_2_-nanobelts caused the biggest toxicological response to THP1 cells, as they are more sensitive to exposure compared to epithelial cell lines RLE-6TN and BEAS-2B [69]. Moreover, it has been reported that this response is specific to the nanobelt form of TiO_2_ [69].

Interestingly, approximately 10% of the pathways in WikiPathways and Reactome can be categorized as toxicity-related, in other words these pathways have at least 50% gene overlap with the GO terms we selected. This highlights the fundamental cellular processes involved but could also indicate a bias in the pathway collections towards these well-studied processes. Nonetheless, these pathways are considered important pathways, hence they are studied well.

**Figure A2:**
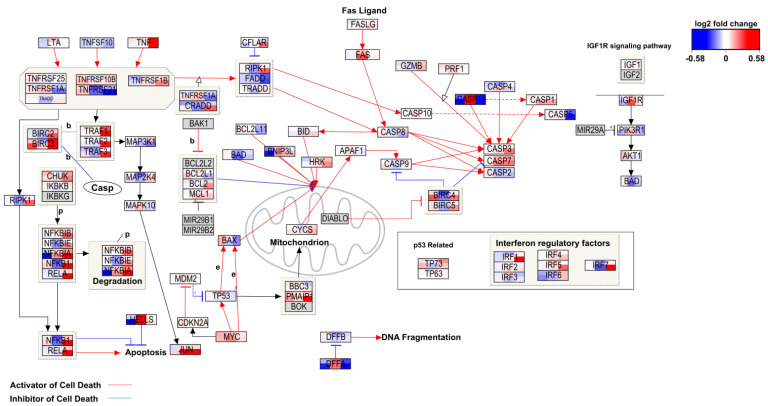
Visualization of log${}_{2}$ fold change on the Apoptosis pathway (wikipathways:WP254) for all six conditions. Cellular models are depicted from left to right as Caco-2/HT29-MTX, SAE, and THP1. The top row depicts the low concentrations whereas the bottom row depicts the high concentrations. Color gradient for differential gene expression after exposure goes from blue (downregulated) via white (not changed) to red (upregulated).

**Figure A3:**
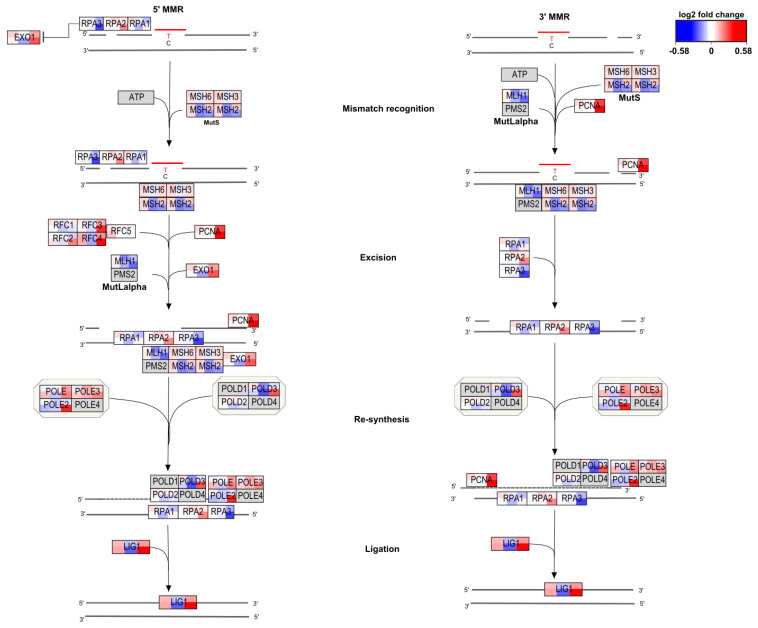
Visualization of log fold change on the DNA Mismatch Repair pathway (wikipathways:WP531) for all six conditions. Cellular models are depicted from left to right as Caco-2/HT29-MTX, SAE, and THP1. The top row depicts the low concentrations whereas the bottom row depicts the high concentrations. Color gradient for differential gene expression after exposure goes from blue (downregulated) via white (not changed) to red (upregulated).

To illustrate how the effects can be studied in more depth we visualized the log fold change of all six conditions on the Oxidative Stress pathway (wikipathways:WP408 [39]), the Apoptosis pathway (wikipathways:WP254 [46]), and the DNA Mismatch Repair pathway (wikipathways:WP531 [51]). This shows that for most genes in these pathways gene expression data are present (see additional Figure A2 and Figure A3).

**Figure 3:**
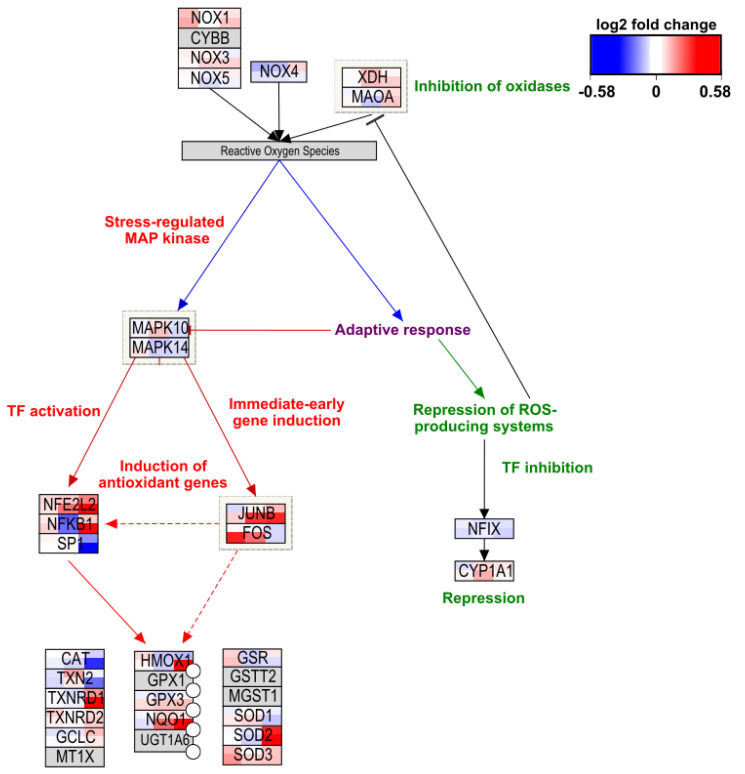
Visualization of log fold change on the Oxidative Stress pathway (wikipathways:WP408) for all six conditions. Cellular models are depicted from left to right as Caco-2/HT29-MTX, SAE, and THP1. The top row depicts the low concentrations whereas the bottom row depicts the high concentrations. Color gradient for differential gene expression after exposure goes from blue (downregulated) via white (not changed) to red (upregulated).

While the Oxidative Stress pathway shown in Figure 3 only shows up to be significantly overrepresented for the THP1 cell line for the high concentration, it is still an interesting pathway to dive deeper into. The NFKB1 gene, which encodes for the NfκB-p105 subunit, has the highest log fold change for the THP1 cell line, high concentration. Additionally, the THP1 cell line, low concentration, shows a positive log fold change as well, whereas the SAE cell line shows negative log fold changes and the Caco-2/HT29-MTX co-culture shows noticeably lower positive log fold changes for both concentrations. Furthermore, the SOD2 gene, which is involved in the conversion of superoxide and protects against oxidative stress, shows a similar pattern [70]. These genes indicate that the THP1 cell line responds to oxidative stress by increasing the expression of protective genes.

Moreover, the Apoptosis pathway shows positive log fold changes of the CASP2 and CASP7 genes, which are involved in apoptosis execution, for the SAE and THP1 cell lines for both concentrations whereas the Caco-2/HT29-MTX co-culture shows no negative or positive log fold change [71,72]. Furthermore, the pathway shows positive log fold changes for the apoptosis-promoting interferon regulatory factors such as IRF4 and IRF5 [73,74,75], which is a similar pattern as described for the CASP2 and CASP7 genes. This could be an indication that apoptosis is stimulated in these cell lines. However, the original study by Tilton et al. does not show a significant decrease in cell viability. The DNA Mismatch Repair pathway shows a positive log fold change for the LIG1 gene, which encodes for DNA ligase 1 for both the Caco-2/HT29-MTX and THP1 cells. This enzyme is involved in both DNA replication and in this context more importantly repair [76]. The aforementioned genes show positive log fold changes for the THP1 cell line throughout the pathway. The increase in expression of these genes could be an indication that DNA mismatch repair is activated due to exposure to TiO_2_-nanobelts.

### 2.9. The Advantage of Our Approach

Enrichment analysis for gene expression data is well-established and can easily be automated to increase reproducibility [77]. The interpretation of the long list of altered, often overlapping, pathways is still a challenge. To mitigate this challenge we proposed an automated approach to filter the pathway enrichment result towards a specific biological focus, in this case toxicity. A more context-specific interpretation is facilitated using GO-term gene sets of interest. The generated pathway-gene networks showcase the connectedness of the processes and provide a more systemic view than looking at individual pathways. The approach yields a fast and easy method with which to select pathways of interest for more detailed scrutiny, as we have shown. While interpretation and comparison between multiple datasets on a process level are still challenging, the automated analysis workflow used supports the exploration of the data [78]. Our approach showed the biological response of the three cellular models on a biological process level, making use of pathways. Using pathways to investigate the response not only highlights biological processes that are affected, but also enables researchers to dive deeper into the pathways on a gene-level. We showed this in our research, where we could easily discuss affected genes in relation to biological processes based on the results we retrieved from our approach. Furthermore, our approach is suitable for quickly retrieving results towards specific biological processes, which will aid researchers in their future research. Moreover, our approach is suitable for quick reanalysis each time new pathway knowledge is discovered, thus pathway databases are updated.

## 3. Conclusions

In this study we investigated the molecular response of three different cell lines to exposure to TiO_2_-nanobelts. Using our process-level analysis based on pathway analysis in combination with the use of gene sets and network visualization, our findings support the results by S.C. Tilton et al. showing that the three cellular models, Caco-2/HT29-MTX, SAE, and THP1, show different toxicity-related responses to the exposure of TiO_2_-nanobelts from resilient Caco-2/HT29-MTX and SAE cells to strongly responding THP1 cells. The latter is not unexpected since the observed effects align with the biological function of these immune cells. Importantly, the approach allowed us not only to find changes in gene expression, but also to find responding molecular pathways via the pathway analysis. Additionally, it allowed us to filter the broad pathway enrichment results to a focused view on the cytotoxic processes affected. The filtering steps we included in our workflow allow a very targeted approach. This allows us to visualize and explore the interactions between responding genes, based on underlying molecular processes in greater detail and in a less time-consuming manner. The approach is suitable for quick reanalysis of datasets each time pathway databases are updated with newly discovered pathway knowledge. Moreover, this versatile approach captured in the R-script can easily be adapted to isolate other processes by using other Gene Ontology terms diverting the focus to other biological processes of interest.

## 4. Materials and Methods

### 4.1. Dataset

In this study, a published and publicly available transcriptomics dataset generated by Tilton et al. [34] was used. The dataset is available from the Gene Expression Omnibus (accession number: GSE42069) [35]. Quality control, data pre-processing, and statistical analysis were performed using scripts from ArrayAnalysis.org [79].

The dataset consisted of 18 samples from three human-like cellular models, i.e., Caco-2/HT29-MTX, SAE, and THP1, which were exposed to either medium (control), 10 μg/mL or 100 μg/mL TiO_2_-nanobelts for 24 h in triplicate. To run the same analysis for the 1 h time point the workflow in the 1 h repository can be used [80]. The number of samples, cellular models, and number of replicates were the same for the 1 h time point compared to the 24 h time point. Culture conditions were kept as identical as possible between the three cell lines for both time points. Details can be found via the original publication by Tilton et al. [34].

Volcano plots depicting differential gene expression were made using the EnhancedVolcano package (version 1.4.0) for R (version 3.6.1) [81,82]. Genes were considered differentially expressed between treated and control when they had an absolute fold change greater than 1.2 (log${}_{2}$ fold change lower than −0.26 or higher than 0.26) and a *p*-value lower than 0.05.

### 4.2. Pathway Analysis

Overrepresentation analysis was performed using the enricher function of the clusterProfiler package (version 3.14.3) [77] for R to identify the molecular changes on a pathway level. The human pathway collection containing 1076 pathways was obtained from WikiPathways (http://www.wikipathways.org (accessed on 28 August 2021), version 20201003, [32]). The Curated and Reactome collections from WikiPathways were included in the analysis. Enrichment analysis was performed for differentially expressed genes using the fold change and *p*-value cutoff as described in the previous section. Additionally, default settings of the enricher function of the clusterProfiler package were used, except *p*-value and q-value (false discovery rate) cutoff were set to 1 to include all results at this stage. This allowed us to later select the desired results based on a *p*-value smaller than 0.05. Minimal gene set size was set to 10 and the maximum gene set size was set to 300.

### 4.3. Toxicity-Related Processes

Within the two human pathway collections used, toxicity-related pathways were identified based on the gene overlap with the toxicity-related gene sets. The overlap was calculated by dividing the number of toxicity-related genes present in the pathway by total number of genes present in the pathway. The gene sets were retrieved from the Gene Ontology (GO, version: release 2020-06) for the following four toxicity-related GO-terms: “apoptotic process” (GO:0006915), “inflammatory response” (GO:0006954), “cellular response to DNA damage stimulus” (GO:0006974), and “response to oxidative stress” (GO:0006979) [83,84]. Associated genes were retrieved using the biomaRt package in R (version 2.42.0, Ensembl Genes 100) [85,86]. Using the GO evidence codes, only genes with experimental evidence or manually curated annotations were included to ensure high confidence that the gene was involved in the specific process (IBA, IC, IDA, IEP, IGI, IMP, IPI, TAS, http://geneontology.org/docs/guide-go-evidence-codes/ (accessed on 28 August 2021), see additional Table A2). Gene overlap between GO terms was visualized using Venny version 2.1.0 [87].

### 4.4. Network Visualization

Altered pathways in the enrichment analysis were filtered for toxicity-related pathways and then visualized as pathway-gene networks to portray the overlap and crosstalk between the pathways. To construct the networks, edge (source: pathway, target: gene) and node (all unique source and target nodes) files were created of the altered pathways. The networks were made using the igraph R package (version 1.2.4.1) [88].

### 4.5. Reproducible Analysis Workflow

The complete analysis is automated in R (version 3.6.3) and can easily be repeated with a new transcriptomics dataset or different selection focus. The R scripts are available on GitHub (https://github.com/laurent2207/TiO2-scripts (accessed on 28 August 2021)) and archived on Zenodo [78].

## Figures and Tables

**Table 1:** Table listing the number of significantly overrepresented pathways, altered toxicity pathways, and the number of altered toxicity pathways for each GO-term.

	Significant	Toxicity	Apoptosis	Inflammation	DNA Damage	Oxidative Stress
**Pathways**	1076	101	66	15	28	2
Caco-2/HT29-MTX low	56	10	5	0	5	0
Caco-2/HT29-MTX high	58	10	10	1	0	0
SAE low	28	3	1	0	2	0
SAE high	50	15	6	1	8	0
THP1 low	38	10	9	4	0	0
THP1 high	201	39	31	9	3	1

## Data Availability

Data analyzed in this paper are from the publicly available dataset generated by Tilton et al. [34] (accession number: GSE42069) [35].

## Abbreviations

The following abbreviations are used in this manuscript:

TiO_2_	Titanium dioxide
SAE	Small airway epithelial cells
THP1	Human monocytic cells
Caco-2	Human epithelial colorectal adenocarcinoma cells
GO	Gene Ontology

## Appendix A

**Table A1:** Table containing the number of pathways related to the four GO-terms. Based on either an overrepresentation analysis (*p*-value < 0.05), 80% gene overlap, 70% gene overlap, 60% gene overlap, or 50% gene overlap.

	Number of Pathways Based on				
	ORA (Adjust *p* Value < 0.05)	80% Gene Overlap	70% Gene Overlap	60% Gene Overlap	50% Gene Overlap
apoptotic process (GO:0006915)	401	11	15	31	66
inflammatory response (GO:0006954)	250	0	0	6	15
DNA damage (GO:0006974)	203	15	20	23	28
oxidative stress (GO:0006979)	205	1	1	1	1

**Table A2:** List of Gene Ontology (GO) evidence annotation codes that were used to remove genes related to the four GO terms with these annotations (bottom part). Additionally, the annotation codes that were present are shown (top part).

Abbreviation	Full Name	Category
IBA	Inferred from Biological aspect of Ancestor	Phylogenetically-inferred annotations
IC	Inferred by Curator	Curator statement evidence codes
IDA	Inferred from Direct Assay	Experimental evidence codes
IEP	Inferred from Expression Pattern	Experimental evidence codes
IGI	Inferred from Genetic Interaction	Experimental evidence codes
IMP	Inferred from Mutant Phenotype	Experimental evidence codes
IPI	Inferred from Physical Interaction	Experimental evidence codes
TAS	Traceable Author Statement	Author statement evidence codes
**Removed annotations**		
ND	No Biological Data Available	Curator statement evidence codes
NAS	Non-traceable Author Statement	Author statement evidence codes
IEA	Inferred from Electronic Annotation	Electronic annotation evidence code
ISS	Inferred from Sequence or structural Similarity	Computational analysis evidence codes
ISO	Inferred from Sequence Orthology	Computational analysis evidence codes
ISA	Inferred from Sequence Alignment	Computational analysis evidence codes
ISM	Inferred from Sequence Model	Computational analysis evidence codes
IGC	Inferred from Genomic Context	Computational analysis evidence codes
RCA	Inferred from Reviewed Computational Analysis	Computational analysis evidence codes
